# Long Non-Coding RNA Associated with Cholesterol Homeostasis and Its Involvement in Metabolic Diseases

**DOI:** 10.3390/ijms21218337

**Published:** 2020-11-06

**Authors:** Kang-Hoon Lee, Hyeon-Ji Hwang, Je-Yoel Cho

**Affiliations:** Department of Biochemistry, BK21 Plus and Research Institute for Veterinary Science, School of Veterinary Medicine, Seoul National University, Seoul 08826, Korea; khlee02@snu.ac.kr (K.-H.L.); hhyunji1027@naver.com (H.-J.H.)

**Keywords:** long non-coding RNA, lncRNA, cholesterol, disease

## Abstract

Cholesterol is an essential cell component that functions to create and maintain all kinds of cell membranes and lipoprotein particles. It is crucial to maintain the proper amount of cholesterol at both the cellular and systemic level. Recently, the importance of cholesterol has been reported not only in various cell development processes but also in the development of diseases. Furthermore, the involvement of long non-coding RNAs (lncRNAs), which are regarded as important epigenetic regulators in gene expression, has also been reported in cholesterol homeostasis. It is thus necessary to summarize the research on lncRNAs related to cholesterol with increased interest. This review organized the role of lncRNAs according to the major issues in cholesterol homeostasis: efflux, metabolism and synthesis, and disease process.

## 1. Introduction

Unlike plants that produce only small amounts of cholesterol called stigmasterol, cholesterol is essential for the survival of animals. Animal cells synthesize cholesterol from simple molecules through complex processes and it can also be absorbed from the outside. Composition and absorption are mutually controlled so that the body can maintain constancy [1,2,3].

Cholesterol is essential for the construction and maintenance of cell membranes and for controlling membrane rigidity [4,5]. Within the cell membrane, cholesterol is also involved with intracellular transport, cell signaling, and neurotransmission [6,7,8]. Additionally, cholesterol is a major precursor molecule to vitamin D, steroid hormones including adrenal hormones, cortisol, and aldosterone, and sex hormones, such as progesterone, estrogen, and testosterone [9]. Systemically, cholesterol is synthesized at different levels depending on the cell type and the function of the organ, but all animal cells synthesize cholesterol as needed. Of the cholesterol synthesized per day, 20 to 25 percent are synthesized in the liver. The synthesized cholesterol is transported through the bloodstream by lipoprotein particles, very-low density lipoprotein (VLDL), low density lipoprotein (LDL), intermediate density lipoprotein (IDL), and high-density lipoprotein (HDL) [10,11]. Further study of cholesterol regulation is required since a high level of cholesterol in the bloodstream is the cause of various diseases, such as coronary heart disease, stroke, peripheral arterial disease, type 2 diabetes, high blood pressure, et cetera [12,13,14,15].

Since the completion of the Human Genome Project in 2004, researchers have assumed that the human genome has about 100,000 protein coding genes and have focused mainly on this area that covers only one percent of the three billion base sequences of human genes [16]. However, in 2012, non-coding RNA was re-evaluated by the ENCODE (Encyclopedia of DNA Elements https://www.encodeproject.org) project, and revealed to have biochemical activity [17]. The findings that these non-coding RNAs are deliberately expressed and serve biological purpose provided new insights into unsolved epigenetic puzzles.

Long non-coding RNA (lncRNA) is classified by having a size bigger than 200 nt. In recent years, the interest in the diverse roles of lncRNAs in post-genetic mechanisms has grown, but research on the structure and function of lncRNAs is still relatively slow progressing. The roles of the major lncRNAs identified so far can be broadly divided into transcriptional regulators, scaffolding protein-protein interaction, and protecting mRNA from miRNA as a competing endogenous RNA [18,19,20]. The major roles of lncRNAs identified so far is largely accomplished through interaction with various proteins and other RNAs at a wide range of stages, such as epigenetic regulation, gene transcription, and post-transcription [21,22,23]. In detail, lncRNAs (1) interact with transcription factors and enhancers to control the expression of various genes; (2) combine with DNA methyltransferase, such as DNMT1, DNMT3A, and DNMT3B to control the methylation status of key molecules in the cell development process; and (3) are also known to work on chromatin structural changes through PRC2 complex formation [24,25,26,27,28]. Additionally, a new mechanism has been reported in which lncRNA acts as cis like XIST, helping the genome take a particular chromosome configuration [29]. Moreover, lncRNAs can act as regulators for epigenetic mechanisms such as DNA methylation and histone transformation. For example, an lncRNA that is expressed in a part of the gene called CEPA has been found to control DNMT1 (DNA methyltransferase 1), which is a well-known DNA methylation regulator [30]. Collectively, lncRNAs can control all the epigenomic phenomena like the conductor of an orchestra. This is possible because the RNA itself is quite dynamic and variable, and flexibly changes depending on the environment. Importantly, lncRNAs also play a crucial role in the development of diseases, such as cancer or neurological diseases as well as aging, but not much research has been conducted on which lncRNAs have functions in each disease [31,32,33,34,35,36].

In this review, we organized lncRNAs and their functions associated with cholesterol synthesis, metabolism, efflux, and related disease processes. This will lead to a better understanding of the epigenetic regulation of cholesterol and associated diseases and provide a new point of view for the development of therapeutic targets.

## 2. LncRNA in Cholesterol Synthesis and Metabolism

Unexpectedly, many lncRNAs with functions in cholesterol metabolism do not directly target genes involved in the pathways for major lipid metabolism, such as triglyceride and cholesterol biosynthesis, and β-oxidation of fatty acids. Instead, numerous lncRNAs have been identified and characterized with the axis of lncRNA-miRNA-target mRNA, which is a well-known process in lncRNA functionality. It has been demonstrated that lncRNA RP5-833A20.1 regulates cholesterol homeostasis by inducing has-miR-382-5p, which results in the downregulation of nuclear factor 1A(NF1A). Interestingly, overexpression of NF1A increased HDL-cholesterol circulation, but reduced LDL-cholesterol and VLDL-cholesterol circulation [37]. Similarly, lncRNA DAPK-IT1 can be targeted by has-miR-590-3p, which regulates the lipoprotein lipase (LPL) gene resulting in increased LPL that might contribute to disease conditions [38]. LncRNA NEAT1 and mir-342-3p are similarly linked together [39]. The involvement of an lncRNA derived from hepatocytes (Lnc-HC) in cholesterol metabolism has been reported for various target molecules and pathways. First, similar to previously mentioned lncRNAs, Lnc-HC can regulate PPARγ-mediated hepatic lipid metabolism via miR-130b-3p, yet lnc-HC has a positive correlation with mir-130b-3p expression [40]. Second, it is known that Lnc-HC forms a complex with hnRNPA2B1 to negatively regulate Cyp7a1 and Abca1 expression that are both implicated in cellular cholesterol excretion [41]. Another lncRNA and hnRNP complex has been reported with LeXis and RALY hnRNP, which are involved in cholesterol biosynthesis [42]. LncRNA HULC has been identified as an overexpressed lncRNA in hepatocellular carcinoma (HCC). HULC decreases miR-9 expression via up-regulation of DNA (cytosine-5-)-methyltransferase 1 (DNMT1). The methylation of a CpG island on miR-9 by DNMT1 decreases its expression, resulting in an increase of peroxisome proliferator-activated receptor alpha (PPARα), also known as NR1C1, which activates the Acyl-CoA synthetase subunit ACSL1. The overexpression of ACSL1 generates sufficient cholesterol to enhance the proliferation of HCC [43].

**Figure 1:**
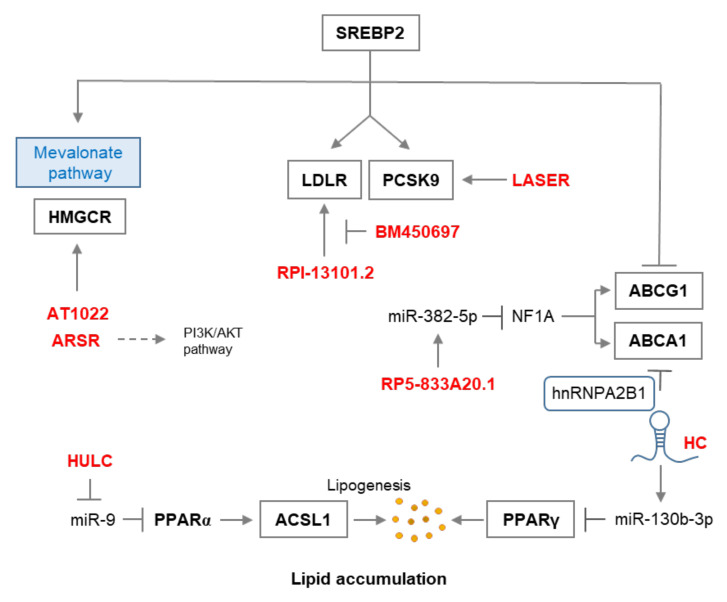
The lncRNAs involved in lipogenesis. Red characters indicate lncRNAs and the boxes indicate the target genes of the lncRNA.

On the other hand, both lncRNA BM450697 and RP1-13D10.2 have been identified as regulators of the low-density lipoprotein receptor (LDLR) family, of which the members are widely involved in cardiovascular disease and lipoprotein homeostasis [44]. BM450697 mechanistically decreases LDLR transcription by blocking interactions with PolII and possibly SREBP1a (sterol regulatory element binding protein 1a transcription factor) at the LDLR promoter [45]. However, RP1-13D10.2 overexpression increases LDLR. Of note, a single-nucleotide polymorphism, rs6924995, within RP1-13D10.2, which was first reported to be associated with low-density lipoprotein cholesterol (LDLC) response to statin, has an association with its expression levels [46]. Additionally, proprotein convertase subtilisin/kexin type 9 (PCSK9), which can bind to LDLR and maintain cholesterol homeostasis in hepatocytes, is known to be regulated by lncRNA, LASER [47]. HMG-CoA reductase (β-hydroxy-β-methylglutaryl-coenzyme A reductase, HMGCR) that catalyses the conversion of HMG-CoA to mevalonate, which is a necessary step in the biosynthesis of cholesterol, is suppressed by cholesterol derived from the internalization and degradation of LDL. Two lncRNAs, AT102202 and LncARSR, have been reported to function by regulating HMGCR expression. Knockdown of AT102202 results in a marked increase of HMGCR expression [48]. However, lncARSR increases the expression of mature SREBP-2, a primary transcription factor of HMGCR, and activates the PI3K/Akt pathway resulting in the promotion of hepatic cholesterol biosynthesis [49]. This positive correlation between lncRNA and target gene is also found between HOXC-AS1 and HOXC6. HOXC-AS1 promotes the expression of the HOXC6 gene and suppresses cholesterol induced by Oxidized-LDL (Ox-LDL) [50]. In addition, although more extensive analysis is necessary, lnc-KDM5D-4 has been suggested to be associated with the accumulation of cholesterol resulting in atherosclerosis and coronary artery disease (CAD) [51]. LncRNAs involved in the lipogenesis pathway are indicated in Figure 1 and summarized in Table 1.

## 3. LncRNAs in Cholesterol Efflux

The term cholesterol efflux refers to the process by which cholesterol is reversely translocated from within the cell to outside the cell and it is an efficient pathway to remove excess cholesterol. Recent extensive studies have revealed several key factors involved in cholesterol efflux, but the relationship between these factors is not clear. The four efflux pathways can be grouped into two passive processes that involve simple diffusion (aqueous diffusion pathway) and facilitated diffusion (SR-BI-mediated pathway) and two active processes that involve members of the ATP-binding cassette (ABC) family of transmembrane transporters, namely ABCA1 and ABCG1 [67]. The regulation of the key factors by lncRNAs, mostly in the active processes, has been investigated in association with atherosclerosis [68].

The regulation of ABCA1 gene expression by lncRNAs is very complicated. Four lncRNAs, DYN-LRB2-2, MALAT1, MeXis and CHROME, have been identified to target ABCA1 through diverse mechanisms. Although the detailed mechanisms are still veiled, it has been revealed that DYN-LRB2-2 promotes cholesterol efflux via negatively regulating the expression of TLR2, which can lead to ABCA1 protein expression [52]. On the other hand, lncRNA MeXis interacts with DDX17, a transcription coactivator that can amplify liver X receptor (LXR) transcription activity on the ABCA1 promoter. The identification of MeXis as an lncRNA modulator of LXR-dependent gene expression expands the understanding of the mechanisms underlying cell type-selective actions of nuclear receptors in physiology and disease [57]. Since one of the most well-studied lncRNAs, MALAT1, is involved in cholesterol efflux via the MALAT1-miR-17-ABCA1 axis, knockdown of MALAT1 promotes cholesterol accumulation in Ox-LDL [59]. CHROME controls cholesterol homeostasis in primates through the regulation of ABCA1 expression. Interestingly, knockdown of CHROME increases the levels of several miRNAs such as miR-27b, miR-33a, miR-33b, and miR-128 [56]. Collectively, ABCA1 expression is regulated exquisitely by these various lncRNAs.

ABCG1 is the other major regulator gene controlling cholesterol efflux and is also targeted by a number of lncRNAs, including ENST00000602558.1, AC096664.3, and TUG1 (taurine upregulated 1). Since ENST00000602558.1 has a negative correlation with ABCG1 expression, ENST00000602558.1 overexpression decreased ABCG1 gene expression, which causes downregulation of ABCG1-mediated cholesterol efflux. Furthermore, the binding of ENST00000602558.1 to p65 to inhibit target gene (ABAG1) expression was found to be a key mechanism [53]. On the contrary, lncRNA AC096664.3 has a positive correlation with ABCG1 expression through the expression of PPARγ [58]. LncRNA TUG1 has a wide association with numerous cholesterol efflux genes such as ApoM, ABCA1, and ABCG1. Overexpression of TUG1 reduces the expression of ApoM, ABCA1, and ABCG1, which results in a down-regulated cholesterol efflux rate. Mechanically, TUG1 competes with FXR1, which negatively targets ApoM to bind to miR-92a [60].

LncRNA ZFAS1 and CDKN2B-AS1 have been identified to target ADAM10 (A Disintegrin and metalloproteinase domain-containing protein 10), which is a sheddase with a broad specificity for peptide hydrolysis reactions. Of note, ADAM10 expression has a negative correlation with cholesterol efflux. ZFAS1 serves as a competing endogenous RNA (ceRNA) to positively regulate ADAM10 and RAB22A expression via sponging miR-654-3p [54]. Oppositely, CDKN2B-AS1 promotes cholesterol efflux via inhibiting ADAM10. Lipid accumulation is reduced and cholesterol efflux is increased with the overexpression of CDKN2B-AS1 or the silencing of ADAM10. Interestingly, CDKN2B-AS1 can bind to DNMT1 to enhance the methylation of the ADAM10 promoter, leading to the promotion of cholesterol efflux [55].

**Figure 2:**
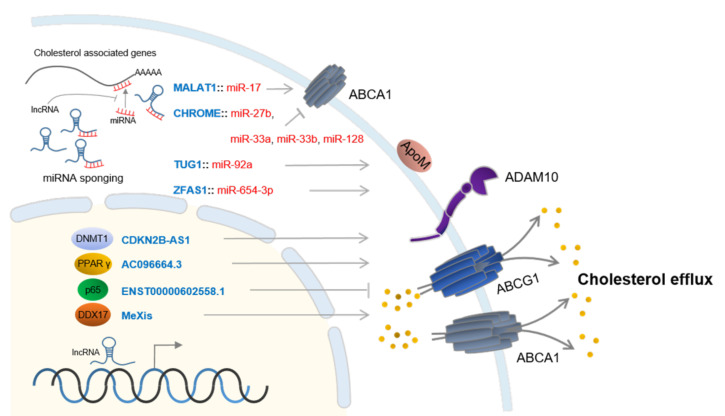
A list of lncRNAs involved in the cholesterol efflux system (i) via recruiting various transcription modulators (DNMT1, PPARγ, p65, DDX17) to ABCA1, ABCG1, ADAM10, and ApoM genes, and (ii) by sponging miRNAs that target ABCA1, ApoM, and ADAM10.

Additionally, cholesterol 7 alpha-hydroxylase (CYP7A1) is also known as an independent contributor to cholesterol efflux. As mentioned before, lnc-HC represses CYP7A1 expression, which results in a decrease of the cholesterol efflux rate [41]. Association of lncRNAs in the cholesterol efflux process was illustrated with their functional target genes and mechanisms in Figure 2. Table 1 summarized the nine representative lncRNAs that have been revealed to be associated with cholesterol efflux.

## 4. Disease Associated Cholesterol Dysregulation via LncRNAs

There are several diseases including atherosclerosis, hepatocellular carcinoma, hypercholesterolemia, and myocardial infarction that are caused by or associated with cholesterol and other lipid fractions [69]. Most studies described earlier in this review have been performed using patient specimens, atherosclerosis model cell lines, such as THP-1 macrophage, hepatocytes, human umbilical vein endothelial cells (HUVEC), vascular smooth muscle cells (VSMC), and knockout mouse models in order to mimic the disease conditions. Consequently, all lncRNAs described in the present review might be closely linked to disease processes, but we summarized several representative studies here that are more focused on lncRNA functions in disease processes.

Hepatic cholestasis is caused by an excessive level of bile acid. Recent studies have shown that SHP (small heterodimer partner, a nuclear receptor) is a key inhibitor of bile acid synthesis and has an important role in the maintenance of normal liver function. LncRNA MEG3 (material representation gene 3) has recently been studied as a potential tumor suppressor and its binding protein PTBP1 (polypyrimidine track binding protein 1 also known as hnRNP) was identified. PTBP1 binds to SHP to disrupt Shp mRNA, which results in a weakened bile acid homeostasis. Contrarily, SHP can also inhibit MEG3 expression by repressing CREB (cAMP response element-binding protein). Collectively, lncRNA MEG3-PTBP1-SHP is an important regulator in both normal liver function and hepatic cholestasis [61]. In addition, lncRNA TUG1 (taurine-up-regulated gene 1) is one of the most well-studied lncRNAs associated with atherosclerosis. TUG1 expression was up-regulated in both in vitro cell lines and an in vivo mouse model. Knockdown of TUG1 inhibited hyperlipidemia and attenuated atherosclerotic lesions in HFD-treated ApoE^−/−^ mice. Technically, TUG1 sponges miR-133a to suppress its activation of FGF1 (fibroblast growth factor 1) resulting in restored FGF1 that overturns the effect of miR-133a on cell proliferation, inflammatory factor secretion, and apoptosis in Ox-LDL-treated macrophages [66].

**Figure 3:**
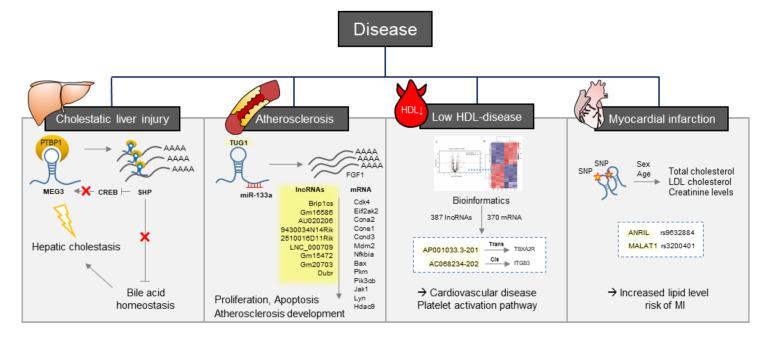
LncRNAs involved in the four major diseases (cholestatic liver injury, atherosclerosis, low HDL-disease, and myocardial infarction) caused by abnormal levels of cholesterol and various lipid fractions. Representative lncRNAs (MEG3, TUG1, AP001033.3, AC068234, ANRIL, and MALAT1) and their mechanisms are illustrated.

Recent bioinformatics studies have identified several lncRNAs that are differentially expressed in the specimen of hypoalphalipoproteinemia (low HDL-cholesterol) disease and myocardial infarction patients as well as in vitro cell line models of diseases that are caused by the disruption of cholesterol homeostasis. Zhang et al. identified a total of 51 lncRNAs and 1730 mRNAs that were significantly dysregulated in the atherosclerosis model. Nine lncRNAs, Brip1os, Gm16586, AU020206, 9430034N14Rik, 2510016D11Rik, LNC_000709, Gm15472, Gm20703, and Dubr were identified as potential biomarkers in macrophage-derived foam cells along with 13 key genes that can influence the degree of cell proliferation and cell apoptosis and the subsequent development of atherosclerosis [63]. A further 381 lncRNAs and 370 mRNAs were reported to be downregulated in patients with low HDL-cholesterol disease. From this result, two lncRNAs AP001033.3–201 and AC068234.2–202, and two potential target genes, integrin beta-3 (ITGB3) and thromboxane A2 receptor (TBXA2R), were identified to be associated with cardiovascular disease and involved in the platelet activation pathway [62]. In the study of coronary artery disease (CAD), lncRNA ENST00000416361 was identified from the comparison of 50 CAD patients with 50 control subjects. SREBP1 and SREBP2 were found to be upregulated in CAD patients and showed positive correlations with the expression of ENST00000416361 [64]. Single nucleotide polymorphisms (SNPs) on the two lncRNAs, ANRIL and MALAT1, can affect the prognosis of myocardial infarction (MI) patients. rs9632884 and rs3200401 SNPs were significantly associated with lipid levels in both controls and MI patients and several SNPs were influenced by sex and age to modify the total cholesterol, low-density lipoprotein cholesterol, and creatinine levels and thus affect the risk of MI [65]. These bioinformatic approaches using clinical specimen and in vitro disease models have successfully suggested candidate lncRNAs involved in cholesterol-associated diseases. However, the results should be confirmed via in vitro and in vivo systems and further functional analysis is mandatory for the use of potential biomarkers and therapeutic targets in the various cholesterol associated diseases (Figure 3). A list of lncRNAs that have been reported in various disease models and patients were summarized in the Table 1.

## 5. Implementation for Diagnosis and Therapeutic Targets

Currently, diagnosis of various cholesterol-related diseases or for the risk of the diseases is mainly done by analyzing the concentrations of lipid fractions such as HDL, LDL, triglycerides, and total cholesterol present in the blood [70,71]. This method can only be performed after 9 to 12 h of fasting to obtain accurate results, and provides very limited information, such as the level of cholesterols [2]. Therefore, if cholesterol-associated lncRNAs are developed as diagnostic biomarkers, more accurate and detailed diagnosis will be possible since lncRNA is found in various body fluids and is stable as mRNA. It also has tissue-specific properties that can be useful clinical indicators when used for diagnosis [72,73,74].

Attempts to use lncRNA as a biomarker for disease diagnosis have been performed in various cancer studies [75,76]. Particularly, the functions and roles of various lncRNAs were revealed during the course of cancer progression including tumorigenesis, metastasis, and resistance to cancer treatment [77,78,79]. Interestingly, some of the cholesterol-related lncRNAs mentioned in this review have also been highlighted in several cancer studies and have been presented as diagnostic biomarkers. HULC (participation in cholesterol synthesis) present in plasma was suggested as a biomarker to identify liver cancer [80]. In addition, the association of MALAT1 (participation in cholesterol efflux) with lung cancer has been studied as a diagnostic marker in numerous papers [81,82]. Similarly, the plasma levels of NEAT1 (participation in cholesterol synthesis) and ANRIL (MI associated lncRNA) were also suggested as biomarkers for non-small cell lung carcinoma (NSCLC) [83,84,85,86]. Furthermore, TUG1 (Atherosclerosis associated lncRNA) has frequently been studied in cancer, and has been shown to recruit specific RNA-binding proteins that can promote cancer expansion and angiogenesis [87]. These results may indicate that lncRNAs might not only have multiple functions in various diseases processes, but also, as has frequently been reported in recent studies, that cholesterol homeostasis is strongly associated with cancer. Collectively, the numerous reports of lncRNAs in cancer means that the development of lncRNA biomarkers for the diagnosis of cholesterol-related disease is very promising.

From a therapeutic point of view, the best ways to prevent or treat cholesterol-related diseases are changes in lifestyle, such as exercise and a general healthy diet [88,89]. However, if abnormal cholesterol levels (mainly high) persist, medication must be taken to lower them. As mentioned earlier, the diagnosis of cholesterol-related diseases is based on the detection of cholesterol-related molecules present in the blood et cetera, and thus the purpose of treatment is to lower the amount of cholesterol to appropriate levels [90]. However, the association of cholesterol to diseases varies widely, so treatments also vary depending on the type and condition of the associated disease. For instance, statins as the main drug, bile oxide, cholesterol absorption inhibitors, et cetera, are all clinically used and have been functionally characterized; statins cut off substances needed to make liver cholesterol, bile oxide increases cholesterol used to make bile acid, and cholesterol absorption inhibitors reduce cholesterol and restrict cholesterol absorption from the small intestine. Recently, drugs that only increase the absorption of LDL cholesterol have also been used. These treatments are often prescribed in combination with each other, with consideration to their actions and side effects [91].

**Figure 4:**
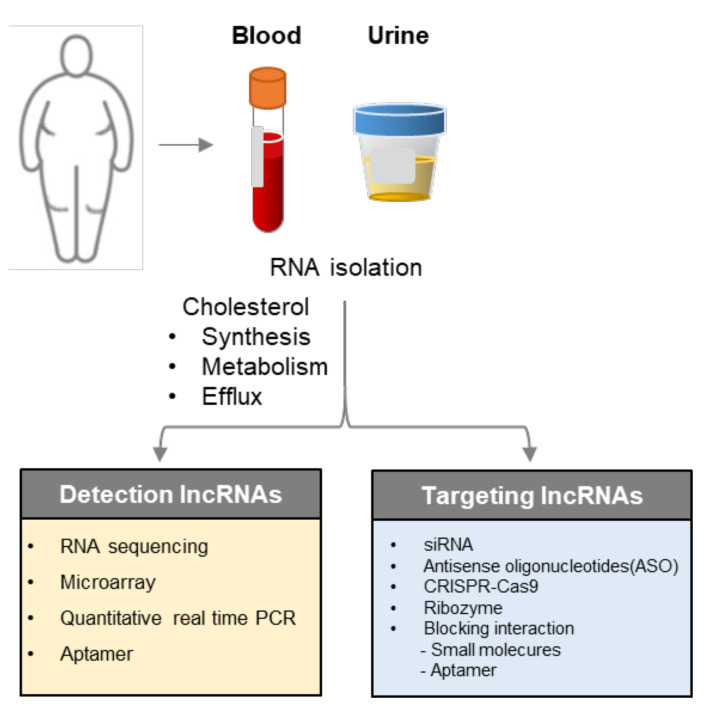
Application of lncRNAs as diagnostic biomarkers and therapeutic targets. LncRNAs in blood or urine specimens can be detected through various methods. The interaction of lncRNAs with target proteins, as well as lncRNAs that are themselves involved in all stages of cholesterol synthesis, metabolism, and efflux will be the candidate therapeutic targets for cholesterol associated diseases.

On the other hand, lncRNAs that are involved in the synthesis, metabolism, and efflux of cholesterol can be used as potential therapeutic targets to maintain cholesterol levels in the human body. In general, RNA interference technology (shRNA, siRNA, anti-sense oligonucleotide (ASO)) is the most promising approach to suppress lncRNA as a target. These approaches have already been proven effective at the cellular level through cancer-related research [92]. For instance, the shRNA knockdown of MALAT1, which was mentioned earlier, significantly reduces the activity of cancer cells [93]. Therefore, if a method for safe nanoparticle-based delivery is developed through further research, treatment with RNA interference technology will become possible [94]. In addition to RNA interference technology, anti-sense oligonucleotide (ASO) based methods are also being studied for more stable and less off-target occurrence. LncRNA MALAT1 targeted ASO has already been developed and its inhibitory effect has been confirmed using various cancer (cervical cancer, lung cancer) animal models [95,96]. In addition to the method that targets lncRNA itself, a method to control the function of lncRNA by inhibiting its interaction with the action protein has also been attempted [21,97]. Small molecule inhibitors mediate the regulation of lncRNAs by blocking the bond of the lncRNA to the interacting protein. A very recent study by Yang et al. used the expression profiles of lncRNAs affected by small molecules from LNCmap to report the global relationships between small molecules and lncRNAs [98]. Moreover, small-molecules were screened to modulate the interaction of lncRNA HOX transcript antisense RNA (HOTAIR) with enhancer of zeste homolog2 (EZH2) [99]. The interaction was inhibited with LSD1 and PRC2 through small-molecule modulators resulting in reduced metastasis of breast cancer [100]. However, it is necessary to further explore and study the lncRNA-protein interaction and pharmacological trends in order to create new small molecule drugs (Figure 4).

## 6. Conclusions

Cholesterol and lipids are very finely regulated in cells and the human body, and their disruption is linked to various diseases. Atherosclerosis is the disease most frequently studied in regard to the association of cholesterol and lncRNA regulation. While it is well known that high cholesterol levels can lead to arteriosclerosis, the association of LDL-cholesterol or other lipids with atherosclerosis remains controversial. Further study of lncRNAs, which play an important role in the foam cell formation process centered on cholesterol-related diseases, can provide important insights to better understand the disease and develop new therapies. A better understanding of the epigenetic regulations that dynamically change according to the surrounding environment grants an opportunity to develop effective treatments. Epigenetic regulation through lncRNA can control target proteins better than conventional drugs. In other words, the study of lncRNAs that are involved in cholesterol control, specifically lncRNAs that directly interact with target genes or proteins, may enable the development of new drugs for the treatment of related diseases. In particular, the latest next-generation sequencing-based big data research has found many lncRNAs associated with various diseases. Yet, molecular biological research is essential to understand the association and actual genetic mechanisms of the various lncRNAs found in this way.

**Figure 5:**
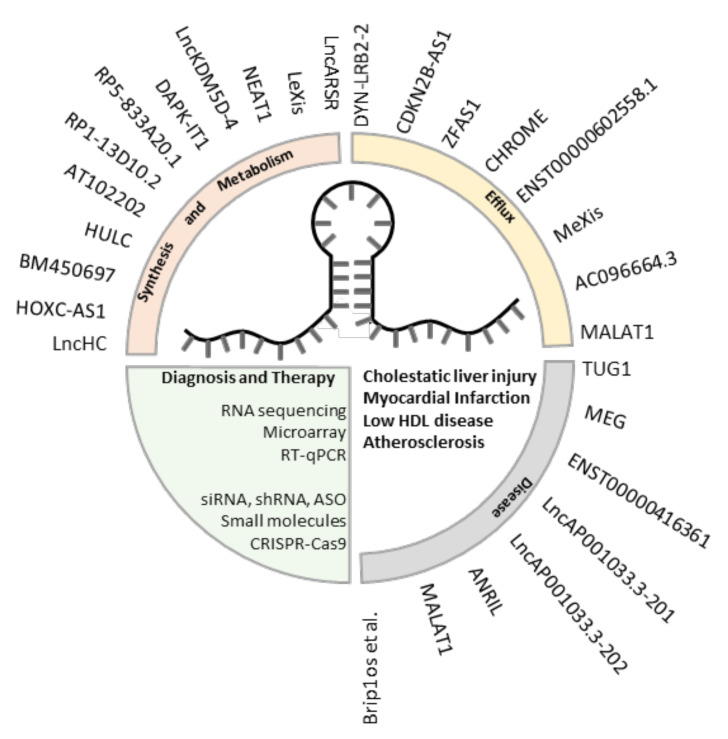
Summary of cholesterol-related lncRNAs. LncRNAs associated with major processes to cholesterol homeostasis, and diseases caused by cholesterol aberrations. Possible approaches to using lncRNAs in clinic are listed.

This review summarized various lncRNAs and their target genes that play a role in the cholesterol homeostasis process (synthesis, metabolism, and efflux). Moreover, the involvement of lncRNAs were presented in various disease conditions (atherosclerosis, myocardial infarction, coronary alternative, low HDL-cholesterol, and hypercholesterolemia). Numerous lncRNAs that have been identified from various studies in diverse diseases are still waiting for further validation to potentially develop more effective diagnosis and therapeutics (Figure 5 and Table 1). Altogether, a deep understanding of these lncRNAs as well as the development of methods for detection and modification are very urgent for clinical application.

## Figures and Tables

**Table 1:** A list of lncRNAs involved in cholesterol metabolism, efflux, and associated disease.

LncRNA	Target	Findings	Ref
**Cholesterol metabolism**			
Lnc-HC	hnRNPA2B1, Cyp7a1, Abca1	Lnc-HC-hnRNPA2B1 complex decreases Cyp7a1, Abca1	[41]
	miR-130b-3p, PPARγ	Negatively regulates PPARγ expression via miR-130b-3p	[40]
BM450697	LDLR	Regulates and local scaffold for LDLR transcription	[45]
HOXC-AS1	HOXC6	Promotes HOXC6	[50]
HULC	ASCL1, PPARA	miR-9/PPARA/ACSL1/cholesterol/RXRA/HULC signaling.	[43]
AT102202	HMGCR	Regulates HMGCR expression	[48]
LASER	PCSK9	Feedback HNF-1α/PCSK9 and LXR dependent pathway	[47]
RP1-13D10.2	LDLR	Regulates LDLR and contributes to LDLC’s response to statin	[46]
RP5-833A20.1	miR-382-5p, NFIA	RP5-833A20.1/miR-382-5p/NFIA pathway	[37]
DAPK-IT1	miR-590-3p, LPL	DAPK1-IT1/hsa-miR-590-3p/LPL axis	[38]
Lnc-KDM5D-4		Key processes related to fatty liver a	[51]
NEAT1	miR-342-3p	Regulates lipid uptake through modulating miR-342-3p	[39]
LeXis	RALY	Binds to RALY to express cholesterol synthetic genes	[42]
LncARSR	SREBP-2, HMGCR	Hepatic cholesterol biosynthesis via Akt/SREBP-2/HMGCR	[49]
**Cholesterol efflux**			
DYN-LRB2-2	TLR2, ABCA1	Upregulates cholesterol efflux by ABCA1 expression	[52]
ENST00000602558.1	ABCG1	Regulates ABCG1 expression through binding to p65	[53]
ZFAS1	miR-654-3p, ADAM10, RAB22A	Elevates ADAM10/RAB22A expression to reduce cholesterol efflux in a miR-654-3p-dependent way	[54]
CDKN2B-AS1	ADAM10	Promotes cholesterol efflux by inhibiting ADAM10	[55]
CHROME	miR-27b, 33a, 33b, 128	Promotes cholesterol efflux and hepatic HDL biogenesis	[56]
MeXis	Abca1	LXR-dependent transcription of Abca1	[57]
AC096664.3	ABCG1, PPAR-γ	Regulates ABCG1 expression via inhibiting PPAR-γ	[58]
MALAT1	miR-17-5p, ABCA1	MALAT1/miR-17-5p/ABCA1 axis	[59]
**Disease**			
TUG1	miR-92a, ApoM, ABCA1, ABCG1	Regulates ApoM, ABCA1 and ABCG1 expression, and attenuates cholesterol efflux via the miR-92a/FXR1 axis	[60]
MEG3	PTBP1	Cholestasis by recruiting PTBP1 to destabilize Shp mRNA	[61]
LncAP001033.3-201	ITGB3, TBXA2R	Associated with low HDL-C disease and could play a role in platelet activation in cardiovascular disease	[62]
LncAC068234.2-202			
Brip1os, Gm16586, AU020206,9430034N14Rik,2510016D11Rik, LNC000709, Gm15472, Gm20703, Dubr	Cdk4, Eif2ak2, Ccna2, Ccne1, Ccnd3, Mdm2, Nfkbia, Bax, Pkm, Pik3cb, Jak1, Lyn, Hdac9	Potential therapeutic targets for atherosclerosis (AS) via an ox-low-density lipoprotein induced macrophage-derived foam cell model	[63]
ENST00000416361	SREBP-1, SREBP-2	Associated with CAD-induced lipid metabolism	[64]
ANRIL, MALAT1	rs9632884 rs1537373, rs619586 rs3200401	Genetic variation of the ANRIL rs9632884 and MALAT1 rs3200401 mediates lipid levels in MI patients	[65]
TUG1	miR-133a, FGF1	TUG1 modulates FGF1 via miR-133a	[66]

## Abbreviations

lncRNA	long non-coding RNA
VLDL	very-low-density lipoprotein
LDL	low density lipoprotein
IDL	intermediate density lipoprotein
HDL	high density lipoprotein
ENCODE	Encyclopedia of DNA Elements
DNMT1	DNA methyltransferase1
NF1A	nuclear factor 1A
LPL	lipoprotein lipase
HCC	hepatocellular carcinoma
PPAR-α	peroxisome proliferator-activated receptor alpha
LDLR	low-density lipoprotein receptor
PCSK9	proprotein convertase subtilisin/kexin type 9
HMGCR	3-hydroxy-3-methyl-glutaryl-coenzyme A reductase
ABC	ATP-binding cassette
ADAM10	A Disintegrin and metalloproteinase domain-containing protein 10
DNMT1	DNA methyltransferase 1
CYP27A1	sterol 27-hydroxylase
HUVEC	human umbilical vein endothelial cells
VSMC	vascular smooth muscle cell
MEG3	material representation gene 3
PTBP1	polypyrimidine track binding protein 1
SHP	small heterodimer partner
CREB	cAMP response element-binding protein
TUG1	taurine-up-regulated gene 1
FGF1	fibroblast growth factor 1
ITGB3	integrin beta-3
TBXA2R	thromboxane A2 receptor
CAD	coronary artery disease
SREBP	Sterol regulatory element binding transcription factor
SNPs	single nucleotide polymorphisms
MI	myocardial infarction
